# Psychosocial Work Environment Explains the Association of Job Dissatisfaction With Long-term Sickness Absence: A One-Year Prospect Study of Japanese Employees

**DOI:** 10.2188/jea.JE20190050

**Published:** 2020-09-05

**Authors:** Akiomi Inoue, Akizumi Tsutsumi, Yuko Kachi, Hisashi Eguchi, Akihito Shimazu, Norito Kawakami

**Affiliations:** 1 Department of Public Health, Kitasato University School of Medicine, Kanagawa, Japan; 2 Faculty of Policy Management, Keio University, Kanagawa, Japan; 3 Department of Mental Health, Graduate School of Medicine, The University of Tokyo, Tokyo, Japan

**Keywords:** absenteeism, job satisfaction, longitudinal studies, psychosocial job characteristics, survival analysis

## Abstract

**Background:**

Using a 1-year prospective design, we examined the association of job dissatisfaction with long-term sickness absence lasting 1 month or more, before and after adjusting for psychosocial work environment (ie, quantitative job overload, job control, and workplace social support) in Japanese employees.

**Methods:**

We surveyed 14,687 employees (7,343 men and 7,344 women) aged 20–66 years, who had not taken long-term sickness absence in the past 3 years, from a financial service company in Japan. The Brief Job Stress Questionnaire, including scales on job satisfaction and psychosocial work environment, was administered, and information on demographic and occupational characteristics (ie, age, gender, length of service, job type, and employment position) was obtained from the personnel records of the surveyed company at baseline (July–August 2015). Subsequently, information on the start dates of long-term sickness absences was obtained during the follow-up period (until July 2016) from the personnel records. Cox’s proportional hazard regression analysis was conducted.

**Results:**

After adjusting for demographic and occupational characteristics, those who perceived job dissatisfaction had a significantly higher hazard ratio of long-term sickness absence than those who perceived job satisfaction (hazard ratio 2.91; 95% confidence interval, 1.74–4.87). After additionally adjusting for psychosocial work environment, this association was weakened and no longer significant (hazard ratio 1.55; 95% confidence interval, 0.86–2.80).

**Conclusions:**

Our findings suggest that the association of job dissatisfaction with long-term sickness absence is spurious and explained mainly via psychosocial work environment.

## INTRODUCTION

Sickness absence is a major public health and economic problem in many countries.^1^^,^^2^ Among others, long-term sickness absence, often defined as sickness absence lasting 4 weeks/1 month or more,^3^ bears high costs for a variety of stakeholders, including employees, employers, insurance agencies, and society at large.^4^^,^^5^ The Organization for Economic Co-operation and Development (OECD) has reported that OECD member countries spend, on average, approximately 1.9% of the gross domestic product (GDP) on sickness absence benefits,^6^ most of which are accounted for by long-term sickness absence.^2^ Furthermore, long-term sickness absence has various adverse effects on employees, such as lower probability of returning to work,^7^^,^^8^ a higher risk of social exclusion,^9^ and mortality.^10^^–^^12^ Therefore, identifying predictors of long-term sickness absence and preventing it are beneficial for both employees and society.

In the occupational health research field, job dissatisfaction (ie, an unpleasant emotion when one’s work is frustrating and blocking the affirmation of their values)^13^ has been attracting attention as a predictor of sickness absence, as well as of poor mental health (ie, anxiety, burnout, depression, and low self-esteem) and physical health (ie, cardiovascular disease and musculoskeletal disorders).^14^ Several prospective studies in European countries have examined the association of job dissatisfaction with sickness absence^15^^–^^24^; the results have been inconsistent, and most of these studies focused mainly on short-term sickness absence lasting from a few days to a few weeks. To date, only three studies focused on long-term sickness absence^16^^,^^21^^,^^22^; two, however, relied on self-reports rather than on personnel records or national register data for measuring sickness absence duration.^21^^,^^22^ This may have led to a less accurate association with job dissatisfaction.^25^ Furthermore, only one study conducted a survival analysis.^23^

In addition to the above, psychosocial work environment may explain the association of job dissatisfaction with sickness absence.^26^ In fact, major psychosocial work environment, such as described in the job demands-control (JD-C) or demand-control-support (DCS) model,^27^^,^^28^ has been associated with job dissatisfaction.^29^^,^^30^ It is also known that poor psychosocial wok environment causes sickness absence.^31^ It might be interesting to know how much unique impact job dissatisfaction has on long-term sickness absence independent of psychosocial work environment, because it would be relevant for developing an effective strategy to prevent long-term sickness absence whether targeting on job dissatisfaction *per se* or psychosocial work environment.

Contrary to European countries, the association between psychosocial work environment, job dissatisfaction, and long-term sickness absence has not been fully examined among Japanese employees. In Japan, approximately 60% of employees reported job-related distress due to psychosocial work environment such as job overload and workplace human relations.^32^ Furthermore, compared to European countries, Japanese employees have been found to have lower levels of job satisfaction,^33^ as well as positive work-related state of mind, such as work engagement.^34^ On the other hand, because the social notion that “not taking time off and working hard are virtues” is still strongly rooted in the Japanese psyche,^35^ taking long-term sickness absence is a serious event for Japanese employees. Therefore, it is extremely valuable to clarify the association of job dissatisfaction with long-term sickness absence and the role of psychosocial work environment in this association among Japanese employees. To date, two cross-sectional studies have reported the association of job dissatisfaction with sickness absence among Japanese employees,^36^^,^^37^ while prospective evidence is lacking and the role of psychosocial work environment in the association is still unclear.

The purpose of the present study was twofold. The first purpose was to examine the prospective association of job dissatisfaction with long-term sickness absence obtained from personnel records in a large sample of Japanese employees, conducting survival analysis. The second purpose was to examine whether psychosocial work environment explains the association of job dissatisfaction with long-term sickness absence. In the present study, we focused especially on financial service employees because they experience increased stress and worries due to greater time pressures, problems with ergonomics, conflicting roles, work demands, and difficult relationships with customers.^38^

## MATERIAL AND METHODS

### Participants

**Figure 1.**
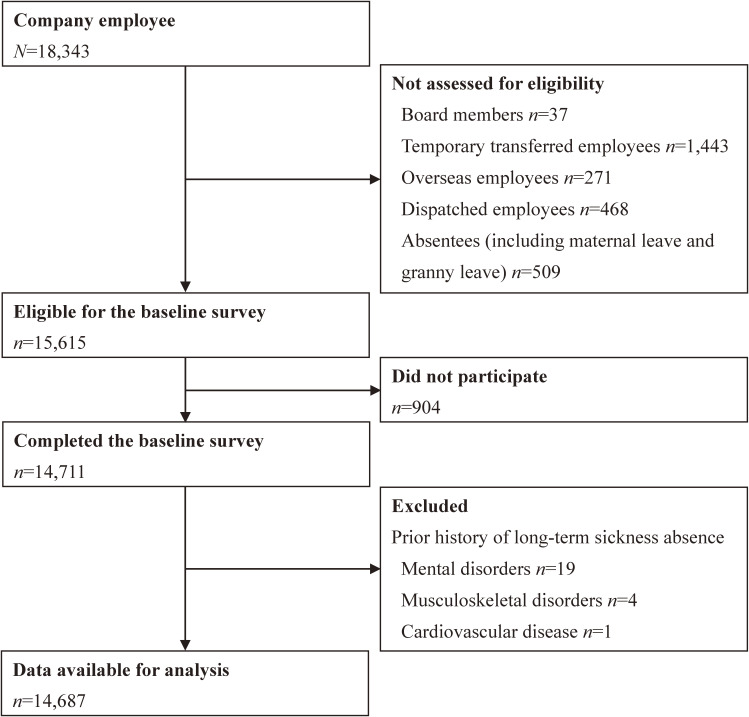
Recruitment and follow-up flow diagram

A 1-year prospective study of employees from a financial service company listed on the major stock exchanges was conducted from July 2015 to July 2016. Information was gathered using a self-administered questionnaire and the personnel records of the surveyed company. At baseline (July–August 2015), all employees, except for board members; temporary transferred, overseas, and dispatched employees; and absentees (*N* = 15,615) were invited to participate in this study; a total of 14,711 employees completed the baseline questionnaire (response rate: 94.2%). After excluding 24 employees who had histories of long-term sickness absence in the past 3 years, 14,687 employees (7,343 men and 7,344 women) aged 20–66 years were followed for 1 year (until July 31st, 2016) (Figure 1). Informed consent was obtained from participants using the opt-out method for the secondary analysis of existing anonymous data. The study procedure was reviewed and approved by the Kitasato University Medical Ethics Organization (No. B15-113).

### Measures

#### Job dissatisfaction

Job dissatisfaction was measured using the Brief Job Stress Questionnaire (BJSQ). The BJSQ has high levels of internal consistency reliability and factor-based validity^39^ and includes a single-item summary measure of job satisfaction (“I am satisfied with my job”). Responses are provided on a four-point Likert scale (1 = *Dissatisfied*, 2 = *Somewhat dissatisfied*, 3 = *Somewhat satisfied*, and 4 = *Satisfied*). Participants were dichotomized into “dissatisfied” (those who answered 1 or 2) and “satisfied” (those who answered 3 or 4) groups.

#### Long-term sickness absence

Information on dates of application for invalidity benefits with medical certification for long-term sickness absence lasting 1 month or more was obtained from the personnel records of the surveyed company. In the surveyed company, it was mandatory for employees to submit medical certification from his/her attending physician to the human resource department when applying for invalidity benefits. Furthermore, the personnel records included information on resignation/retirement date. Based on this information, those who resigned/retired from the surveyed company during the follow-up period were treated as censored cases. The follow-up began on the date of response to the BJSQ and ended at the start date of long-term sickness absence (ie, the date of application for invalidity benefits), the resignation/retirement date, or July 31st, 2016, whichever came first.

#### Psychosocial work environment

For psychosocial work environment, we examined quantitative job overload, job control, and workplace social support, based on the JD-C or DCS model.^27^^,^^28^ These were measured using the BJSQ introduced above. The BJSQ includes three-item quantitative job overload, job control, supervisor support, and coworker support scales. The answers are provided on a four-point Likert scale (1 = *Not at all*, 2 = *Somewhat*, 3 = *Moderately so*, and 4 = *Very much so* for quantitative job overload and job control; 1 = *Not at all*, 2 = *Somewhat*, 3 = *Very much*, and 4 = *Extremely* for supervisor support and coworker support), with the scores of each scale ranging from 3–12. For workplace social support, total scores for supervisor support and coworker support were calculated (score range: 6–24). In this sample, the Cronbach’s alpha coefficients were 0.78, 0.70, and 0.88 for quantitative job overload, job control, and workplace social support, respectively.

#### Covariates

Covariates included demographic and occupational characteristics, all of which were obtained from the personnel records of the surveyed company. Demographic characteristics included age and gender. Age was used as a continuous variable. Occupational characteristics included length of service, job type, and employment position. Length of service was used as a continuous variable. Job type was classified into four groups: sales, claims service, administrative, and others. Employment position was classified into five groups: manager, staff, senior employee, temporary employee, and others.

### Statistical analysis

We first conducted a descriptive analysis using Student’s *t* test or Fisher’s exact test to compare the demographic and occupational characteristics and the scale scores between the satisfied and dissatisfied groups. Afterwards, the cumulative hazard of long-term sickness absence was plotted as Kaplan-Meier curves and the log-rank test was conducted to compare the hazard functions between the satisfied and dissatisfied groups. Finally, using the satisfied group as a reference, Cox’s proportional hazard regression analysis was conducted to estimate the hazard ratio (HR) and its 95% confidence interval (CI) of the incidence of long-term sickness absence during the follow-up period in the dissatisfied group. In the series of analyses, we first adjusted for demographic characteristics (ie, age and gender) (model 1). Subsequently, we incrementally adjusted for occupational characteristics (ie, length of service, job type, and employment position) (model 2) and psychosocial work environment (ie, quantitative job overload, job control, and workplace social support) (model 3). For model 3, overcontrol bias due to common method variance might occur since the present study measured job dissatisfaction and psychosocial work environment simultaneously with the same self-administered questionnaire (ie, the BJSQ). Therefore, to test the presence of overcontrol bias due to common method variance, Harman’s single-factor test^40^ was conducted by entering items for job dissatisfaction, quantitative job overload, job control, and workplace social support (ie, a total of 13 items) into the unrotated principal component analysis. Furthermore, as sub-analyses, the log-rank test and the Cox’s proportional hazard regression analysis were conducted by gender because men and women are exposed to different work environment in Japan. The level of significance was 0.05 (two-tailed). The statistical analyses were conducted using IBM^®^ SPSS^®^ Statistics Version 23.0 for Windows (IBM Corp., Armonk, NY, USA).

## RESULTS

Table 1 shows the detailed characteristics of the participants in the satisfied and dissatisfied groups. Compared to the satisfied group, the dissatisfied group was significantly younger, had a greater proportion of women, claims service, staff, and temporary employees, and perceived significantly higher levels of quantitative job overload and lower levels of job control and workplace social support.

**Table 1.** Demographic and occupational characteristics and scale scores among employees who participated in the study

	Satisfied group (*n* = 11,139)		Dissatisfied group (*n* = 3,548)		*P* value^a^
	
	Mean (SD)	*n* (%)	Mean (SD)	*n* (%)	
Age, years	41.6 (12.4)		41.1 (12.2)		0.027
Gender					<0.001
Men		6,081 (54.6)		1,262 (35.6)	
Women		5,058 (45.4)		2,286 (64.4)	
Length of service, years	12.7 (10.3)		12.1 (9.88)		0.003
Job type					<0.001
Sales		5,360 (48.1)		1,676 (47.2)	
Claims service		3,829 (34.4)		1,426 (40.2)	
Administrative		1,941 (17.4)		442 (12.5)	
Others		9 (0.1)		4 (0.1)	
Employment position					<0.001
Manager		2,086 (18.7)		257 (7.2)	
Staff		6,590 (59.2)		2,457 (69.3)	
Senior employee		465 (4.2)		92 (2.6)	
Temporary employee		1,989 (17.9)		738 (20.8)	
Others		9 (0.1)		4 (0.1)	
Quantitative job overload (3–12)	9.10 (1.86)		10.1 (1.95)		<0.001
Job control (3–12)	8.35 (1.62)		6.79 (1.78)		<0.001
Workplace social support (6–24)	17.6 (3.56)		14.2 (3.46)		<0.001

SD, standard deviation.

^a^Student’s *t* test and Fisher’s exact test were used for the continuous and categorical variables, respectively.

**Figure 2.**
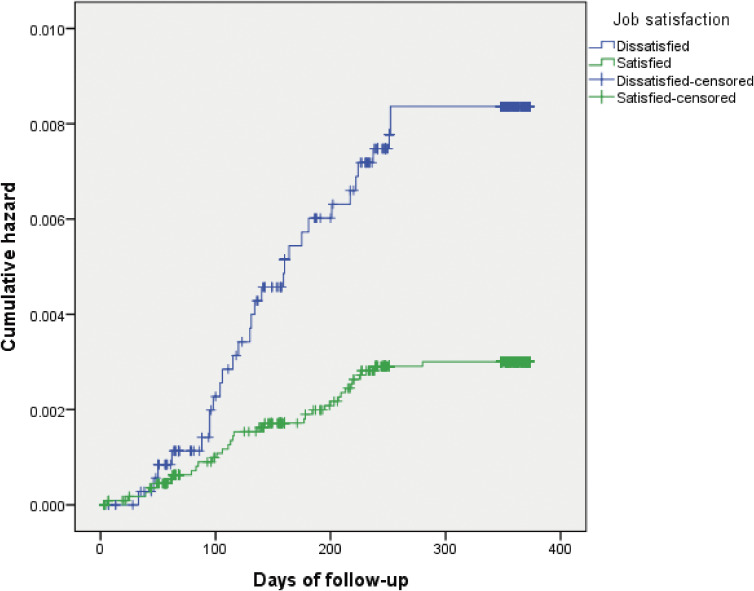
Cumulative hazard of long-term sickness absence among the dissatisfied group compared to the satisfied group

Figure 2 shows the Kaplan-Meier curves for the cumulative hazard of long-term sickness absence among the dissatisfied group compared to the satisfied group. The log-rank test showed that the dissatisfied group had a significantly higher incidence rate of long-term sickness absence compared to the satisfied group (*P* < 0.001).

Table 2 shows the results of the Cox’s proportional hazard regression analysis. During 5,258,910 person-days (mean: 358 days, range: 3–373 days), 62 employees (32 men and 30 women) took long-term sickness absence (mental disorders: 51 cases, musculoskeletal disorders: 6 cases, cerebrovascular disease: 3 cases, and cardiovascular disease: 2 cases). After adjusting for demographic and occupational characteristics (models 1 and 2), the dissatisfied group had a significantly higher HR of long-term sickness absence than the satisfied group (HR 3.00; 95% CI, 1.80–5.00 and HR 2.91; 95% CI, 1.74–4.87 for models 1 and 2, respectively). However, after additionally adjusting for psychosocial work environment (model 3), this association was weakened and no longer significant (HR 1.55; 95% CI, 0.86–2.80).

**Table 2.** Association of job dissatisfaction with long-term sickness absence among Japanese employees: Cox’s proportional hazard regression analysis (7,343 men and 7,344 women)

	Person-days	Number of events	Rate/100,000 person-days	Hazard ratio (95% confidence interval)		

				Model 1^a^	Model 2^b^	Model 3^c^
Main analysis						
Satisfied	3,998,784	33	0.83	1.00	1.00	1.00
Dissatisfied	1,260,126	29	2.30	3.00 (1.80–5.00)	2.91 (1.74–4.87)	1.55 (0.86–2.80)

Gender-stratified analysis						
Men						
Satisfied	2,172,019	17	0.78	1.00	1.00	1.00
Dissatisfied	443,864	15	3.38	4.20 (2.08–8.46)	4.13 (2.03–8.42)	2.00 (0.86–4.63)
Women						
Satisfied	1,826,765	16	0.88	1.00	1.00	1.00
Dissatisfied	816,262	14	1.72	2.05 (0.99–4.21)	1.97 (0.95–4.06)	1.14 (0.50–2.63)

^a^Adjusted for age (and gender).

^b^Additionally adjusted for length of service, job type, and employment position.

^c^Additionally adjusted for quantitative job overload, job control, and workplace social support.

For the Harman’s single-factor test, three factors with eigenvalues greater than 1.0 were extracted and the first (largest) factor did not account for a majority of the variance (32.7%), indicating that overcontrol bias due to common method variance was not of great concern.

When we conducted the gender-stratified analysis, similar tendency to the main analysis was observed among both genders while statistical significance was marginal for the log-rank test (*P* = 0.063) and for models 1 and 2 of the Cox’s proportional hazard regression analysis among women (Table 2).

## DISCUSSION

The present study demonstrated that after adjusting for demographic and occupational characteristics, those who perceived job dissatisfaction had a significantly higher risk of long-term sickness absence during the 1-year follow-up period than those who perceived job satisfaction. After additionally adjusting for psychosocial work environment based on the JD-C or DCS model, the risk was no longer significant.

Job dissatisfaction was significantly associated with a higher risk of long-term sickness absence after adjusting for demographic and occupational characteristics. This finding is consistent with previous prospective studies in European countries (ie, Norway and the Netherlands) that have reported a significant association of job dissatisfaction with long-term sickness absence in the crude model,^22^ as well as after adjusting for demographic and occupational characteristics (eg, age, gender, education, and affiliation).^16^^,^^21^ Using personnel records to measure long-term sickness absence and conducting a survival analysis, the present study expanded this evidence into other than European countries.

After additionally adjusting for psychosocial work environment based on the JD-C or DCS model, the association of job dissatisfaction with long-term sickness absence was weakened and no longer significant. This is consistent with previous studies in that a significant association of job dissatisfaction with sickness absence (including both short-term and long-term ones) was not observed when psychosocial work environment was included in the model.^16^^,^^17^^,^^20^ Our findings suggest that the association of job dissatisfaction with long-term sickness absence is explained mainly by psychosocial work environment and that improving psychosocial work environment is effective for the prevention of long-term sickness absence. However, although not statistically significant, the fully adjusted HR of job dissatisfaction was still approximately 1.5; therefore, there may be a unique effect of job dissatisfaction on long-term sickness absence independently of psychosocial work environment. Future research should examine more precisely the association between psychosocial work environment, job dissatisfaction, and sickness absence.

Possible limitations of the present study should be considered. First, our sample was recruited from one financial service company in Japan; therefore, our findings should be interpreted with caution in light of limited generalizability. Second, job dissatisfaction was measured using a single-item question, which may limit its measurement validity; however, some researchers have argued that single-item questions are preferred to measure overall job dissatisfaction because differences in individual scores are lost in the total mean scores of multi-item questions.^41^^,^^42^ Third, some employees may have transferred to another department in the surveyed company, which may have influenced job dissatisfaction and masked the true association; nevertheless, the frequency of transfer may not have been so high at 1-year follow-up. Finally, although some previous studies focused on workplace-level (in addition to individual-level) job dissatisfaction to examine its association with sickness absence,^19^ the present study could not take workplace-level job dissatisfaction into account due to a lack of information on the departments to which the individual participants belonged.

In conclusion, the present study provided evidence that the association of job dissatisfaction with long-term sickness absence lasting 1 month or more is spurious and explained mainly via adverse psychosocial work environment. More detailed underlying mechanisms in the association between psychosocial work environment, job dissatisfaction, and sickness absence can be explored using mediation analysis.
